# Synthesis of Submicron CaCO_3_ Particles in 3D-Printed Microfluidic Chips Supporting Advection and Diffusion Mixing

**DOI:** 10.3390/mi15050652

**Published:** 2024-05-15

**Authors:** Ivan Reznik, Ekaterina Kolesova, Anna Pestereva, Konstantin Baranov, Yury Osin, Kirill Bogdanov, Jacobus Swart, Stanislav Moshkalev, Anna Orlova

**Affiliations:** 1 International Research and Education Center for Physics of Nanostructures, ITMO University, Saint Petersburg 197101, Russia; e.p.kolesova@gmail.com (E.K.); kirw.bog@gmail.com (K.B.); 2 Faculty of Electrical Engineering and Computing, University of Campinas, Campinas 13083-970, Brazil; jacobus@unicamp.br; 3 Research Center for Translation Medicine, Sirius University, Sochi 354349, Russia; 4 International Laboratory Hybrid Nanostructures for Biomedicine, ITMO University, Saint Petersburg 199034, Russia; anna.pestereva.90@mail.ru (A.P.); baranov.const@mail.ru (K.B.); a.o.orlova@gmail.com (A.O.); 5 Laboratory for Scientific Restoration of Precious Metals, The State Hermitage Museum, Saint Petersburg 191186, Russia; yury.osin@gmail.com; 6 Center for Semiconductor Components and Nanotechnology, University of Campinas, Campinas 13083-870, Brazil; stanisla@unicamp.br

**Keywords:** vaterite, microfluidic synthesis, additive manufacturing, nanoparticles, one-phase synthesis

## Abstract

Microfluidic technology provides a solution to the challenge of continuous CaCO_3_ particle synthesis. In this study, we utilized a 3D-printed microfluidic chip to synthesize CaCO_3_ micro- and nanoparticles in vaterite form. Our primary focus was on investigating a continuous one-phase synthesis method tailored for the crystallization of these particles. By employing a combination of confocal and scanning electron microscopy, along with Raman spectroscopy, we were able to thoroughly evaluate the synthesis efficiency. This evaluation included aspects such as particle size distribution, morphology, and polymorph composition. The results unveiled the existence of two distinct synthesis regimes within the 3D-printed microfluidic chips, which featured a channel cross-section of 2 mm^2^. In the first regime, which was characterized by chaotic advection, particles with an average diameter of around 2 μm were produced, thereby displaying a broad size distribution. Conversely, the second regime, marked by diffusion mixing, led to the synthesis of submicron particles (approximately 800–900 nm in diameter) and even nanosized particles (70–80 nm). This research significantly contributes valuable insights to both the understanding and optimization of microfluidic synthesis processes, particularly in achieving the controlled production of submicron and nanoscale particles.

## 1. Introduction

Drug delivery for cancer treatment represents a pivotal research aimed at enhancing therapeutic efficacy while minimizing systemic side effects [1,2,3]. The Enhanced Permeability and Retention (EPR) effect, which is intrinsic to the unique characteristics of induced tumor vasculature, including hypoxia, allows for a preferential accumulation of nanoparticles within a tumor microenvironment [4,5]. Using nanoparticle-based drug delivery systems capitalizes on the EPR effect to achieve targeted drug delivery, thereby optimizing drug concentrations at the tumor site and mitigating off-target effects [6]. Despite significant advancements, only a small number of nanoparticles in medicine reach the stage of clinical trials and receive approval [7,8]. Micro- and nanoparticle platforms based on calcium carbonate (CaCO_3_) promise advancing drug delivery in cancer therapy [9,10]. To minimize the off-targeting effect, controlled-release systems responsive to external conditions like temperature, enzyme activity, or pH emerge as a viable approach [9,11]. The capability of degrading porous CaCO_3_ nanoparticles layer by layer as the pH level decreases provides control over the release rate of molecules from the subsequent layers of the porous matrix, as well as the oxygen enrichment time for a further excitation in cancer cells/tumors [9,12]. Additionally, the particle porosity can also control the rate of CaCO_3_ degradation and payload release. Traditionally used techniques produce micron-sized particles, while reducing the size of nanocarriers can significantly increase the drug delivery efficiency by improving biodistribution, circulation time, and cellular internalization [13,14,15,16,17].

Microfluidic technologies are well-established and widely used for the chemical synthesis of nanomaterials in various fields, such as chemistry, materials science, biomedicine, and environmental sciences [18,19,20,21,22]. Microfluidic systems offer significantly higher surface-to-volume ratios compared to conventional flask-based systems, thus resulting in better control over heat and mass transfer rates. Literature analysis shows that microfluidic synthesis outperforms flask-based synthesis in terms of controllability, monodispersity, reproducibility, reagent efficiency, and impurity minimization due to better control over nanocrystal growth processes [23,24].

Previous attempts to optimize the crystallization of CaCO_3_ in microfluidic devices have involved changes in microfluidic device topology and use of different flow regimes (continuous-flow mixing, droplet microreactors, and pore networks). These methodologies include the use of various types of flows—turbulent, laminar, and mixed—as well as different configurations of microfluidic chips, such as porous chips, segmented chips, and continuous flow chips. Porous chips allow for the synthesis of a wide range of particles, ranging from tens of nanometers to several micrometers in diameter, but with limited control over the end product [25,26,27]. In the case of segmented chips, nano-sized particles with a narrow size distribution are obtained; however, their maintenance requires more effort due to the use of a multiphase medium and/or various surfactants for droplet generation [28,29,30]. Continuous flow chips typically generate predominantly micron-sized particles with a wide size distribution and a monocrystalline structure [31,32].

Additive manufacturing, or 3D printing, is increasingly used for making microfluidic devices. Techniques like stereolithography allow for the precise fabrication of intricate geometries for PDMS master molds [8,10]. Advantages include rapid prototyping, customization, and integration of multiple functions. It enables the implementation of unconventional structures not feasible with traditional methods [33]. With material and technique advancements, additive manufacturing accelerates microfluidic device innovation for diverse applications such as diagnostics, synthesis, and monitoring.

In this paper, we present a simple route for the synthesis of submicron CaCO_3_ particle growth in vaterite form. The key to our study is the design and 3D printing of microfluidic chips with 2 mm^2^ and 0.5 mm^2^ channel cross-sections. Through CFD simulations, we conducted an in-depth analysis of synthesis dynamics, thereby revealing two distinct regimes of CaCO_3_ precipitation. The first regime, chaotic advection, produced micron-sized particles, while the second regime, diffusion, resulted in a synthesis of submicron particles. The design in this work system facilitated the synthesis of both micron- and submicron-sized CaCO_3_ particles, thereby showcasing the efficiency of additive manufacturing in microfluidic synthesis.

## 2. Materials and Methods

### 2.1. Materials Used

The materials utilized in this study were as follows: calcium chloride (Sigma Aldrich, St. Louis, MO, USA), sodium carbonate (Vekton, Saint-Petersburg, Russia), deionized water, ethylene glycole (Ekos 1, Moscow, Russia), ethanol, and photopolymer resin (Anycubic, Hong Kong, China). The water for the experiments was purified using a Millipore system (Merck Millipore, Moscow, Russia). The rest of the chemicals were used without further purification.

### 2.2. Instruments and Techniques for Investigating the Physical, Chemical, and Optical Properties of Samples

The size distribution of synthesized CaCO_3_ particles were analyzed through scanning electron microscopy (SEM). SEM measurements were conducted using a scanning electron microscope SU 7000 HITACHI equipped with the EDS detectors BRUKER (XFlash FlatQUAD 5060F (Bruker, Hitachi, Japan)). The specimen was deposited on a silicon substrate with a subsequent application of a 7 nm conductive layer of Cr. The application of the conductive layer was performed using the cathodic sputtering method in the vacuum setup EMACE600—LEICA. For the analysis of the morphology and sizes of nanoparticles, both LD and UD detectors were employed (LD—lower SE detector and UD—upper intra-lens SE detector). The accelerating voltage was set at 5 keV. The elemental quantitative analysis and construction of element distribution maps of the nanoparticles were carried out at an accelerating voltage of 15 keV using BRUKER XFlash 6160 EDS detectors. The probing depth at 15 keV was 1 micron. The morphology of the synthesized particles was analyzed with Raman spectroscopy. The Raman spectra were obtained on an InVia micro-Raman spectrometer (Renishaw, UK). The measurements were carried out under excitation by an Ar+ laser with a wavelength of 514 nm. The InVia spectrometer operates under backscattering conditions and has a Leica microscope with an ×50 objective, Na = 0.75, and a multichannel detector (CCD camera) cooled to −70 °C. The area of irradiation with an exciting light is 2 μm.

### 2.3. Microfluidic Chip Fabrication

For the synthesis of CaCO_3_ microspheres, microfluidic chips were prepared according to the procedure described in the work already published by our lab [34]. The microfluidic chip consisted of [N × M] matrix-replicated basic cells, two inlets, and one outlet. Each basic cell was characterized by three parameters: channel width (Ch-W), ranging from 0.5 to 2 mm; a wall width (W-W) of 0.8 mm; and a channel height (Ch-H) of 1 mm. Connector cells were used to link the columns of the basic cells. Based on this logic, a script was developed for the parametric generation of microfluidic chips using the OpenScad software package. To create physical replicas of the chips using additive manufacturing methods, the chip design was subtracted from a solid block, thus forming the microfluidic channel cavities. The direct printing of microfluidic chips was carried out using the Anycubic Photon Mono (Anycubic, Hong Kong, China) photopolymer 3D printer utilizing generic photopolymer resin (Anicubic, Hong Kong, China). The 3D printer resolution was 50 μm for both the XY plane and Z direction.

The printing parameters for the photopolymer resin were as follows: the thickness of one layer was 50 μm, and each layer was exposed to UV light (405 nm) for 2 s. After printing, a significant amount of unpolymerized resin remained within the chip channels and on its surface. To remove this residue, the chip was placed in an ultrasonic bath filled with isopropyl alcohol for 5–10 min. Subsequently, the internal chip channels were rinsed with pure isopropyl alcohol using a syringe pump connected to the chip’s outlet.

The connection of the syringe pumps to the microfluidic chip was achieved using Teflon tubes with internal and external diameters of 1 mm and 1.5 mm, respectively, and steel adapters with internal and external diameters of 0.9 mm and 1.1 mm, respectively. The chip connection process was performed in two stages. In the first stage, a steel adapter was inserted at one end into a Teflon tube and into an inlet or outlet hole at the other end of the microfluidic chip. In the second stage, to ensure chip sealing, all connections were coated with multiple layers of photopolymer resin, which was followed by UV light exposure at a wavelength of 405 nm for 10–30 s to polymerize the joints.

### 2.4. Computational Fluid Dynamics Simulations

The 2D chip designs were created using the OpenScad software 2021.01 package, which were then subsequently imported into COMSOL Multiphysics 5.2 as a foundation for the computational fluid dynamics simulations. The simulations utilized the “Laminar flow (spf)” and “Transport of Diluted Species (tds)” interfaces. The solver was configured to handle time-dependent equations. To optimize the computational efficiency in the CFD process, the mesh size for microfluidic channels was set to 10 μm.

The obtained results were employed to assess the variation in the fluid mixing dynamics between two distinct chip designs, each having channel widths of 0.5 and 2 mm, respectively. The model initially contained water, and reagent solutions were introduced from the inlet at the microchannel’s edge with a flow rate of 1 μL/s. A zero-gauge pressure condition was established at the outlet.

For the simulation of the CaCl_2_ and Na_2_CO_3_ reagent solutions, both inlets were configured to introduce water, and two separate aqueous phases were defined with viscosities, densities, and diffusion constants corresponding to 0.33 M solutions. The resulting phase composition was graphically represented. Table 1 provides a summary of all the constants utilized in the CFD calculations.

### 2.5. Synthesis of CaCO_3_ Particles

In all experiments, the aqueous solutions of CaCl_2_ and Na_2_CO_3_ with concentrations of 0.33 mol/L each were used as precursors for the following reaction equation:

(1) $${CaCl}_{2}+{Na}_{2}{CO}_{3}\to {CaCO}_{3}.$$

The components were mixed in a 1:1 ratio either in an Eppendorf tube using a magnetic stirrer for 100 s or in a microfluidic chip for the same amount of time. The total volume if the reagent mixture was used for each synthesis type was set to 200 μL. The mixing time duration in the microfluidic chip was controlled by using a microfluidic chip with an inner volume of 100 μL and a speed of reagent flow set to 0.001 mL/s. After mixing, the reagent mixture and resulting calcium carbonate particles were diluted 2, 5, and 10 times using distilled water. These solutions, along with the undiluted mixture, were then cleansed twice from unreacted reagents by centrifugation at a speed of 5000 rpm for one minute. Subsequently, the precipitate was redissolved in 100 μL of water and drop-casted onto the surface of a glass slide for further analysis.

## 3. Results and Discussion

### 3.1. Passive Inhibition of CaCO_3_ Synthesis

**Figure 1:**
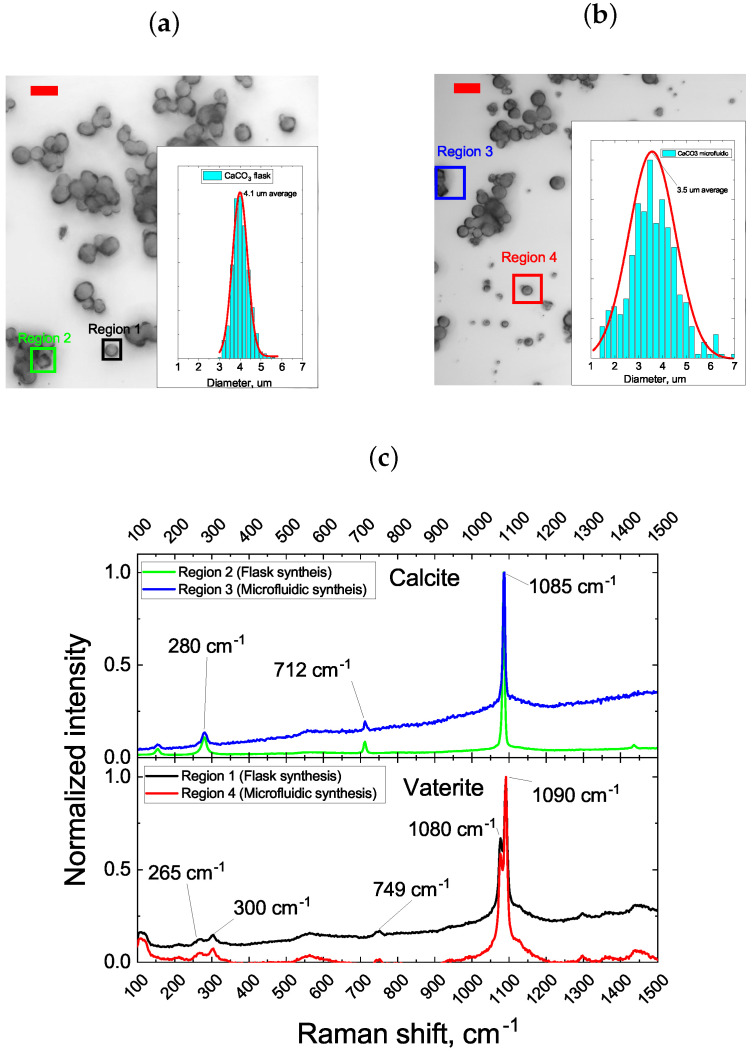
(**a**,**b**)−Microscopic images of the CaCO_3_ particles synthesized in a flask and microfluidic chip, respectively. Bar length on the images is 5 μm. The inset–size distribution of the respective synthesis batch of CaCO_3_ particles. (**c**) Raman spectra of the CaCO_3_ particles taken from the sites designated on microscope images.

The synthesis of calcium carbonate particles is widely used for its simplicity and rapidity, requiring only the mixture of reactants for the initiation and continuation of the reaction process [35,36]. These minimal requirements make the transition from bulk flask synthesis to continuous microfluidic synthesis relatively straightforward. Initially, we conducted a comparative analysis of the size distribution and morphology of microparticles synthesized using both the flask and microfluidic methods. To ensure comparability, synthesis conditions were closely matched: both approaches employed the same reagent concentration of 0.33 M and a synthesis duration of 100 s. In the case of microfluidic synthesis, the reaction time was governed by the fluid velocity required to traverse the inner volume of the microfluidic chip within the specified timeframe (exact parameters and principal schematic of the microfluidic chip used in this experiment are given in Section 2). Figure 1 displays the microscope images, size distribution profiles, and Raman spectra of the resultant particles.

**Figure 2:**
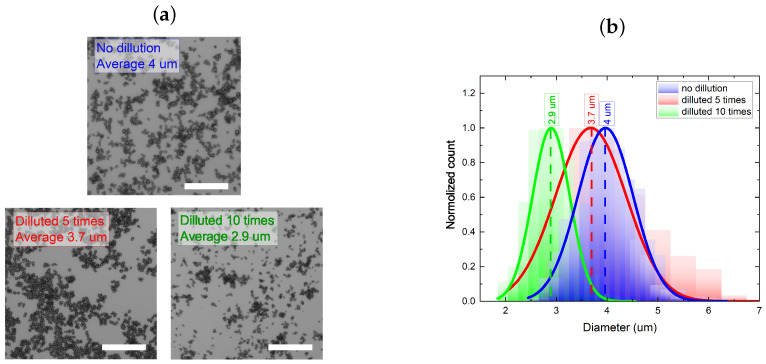
Confocal images (**a**) and the normalized size distribution plots (**b**) of CaCO_3_ particles, which were diluted by a factor of 5 (red curve), 10 (green curve), as well as undiluted (blue curve) prior to precipitation from the precursor mixture. To determine the peak position of the particle size distribution, the size distribution curves were fitted with a Gaussian function. A 20-micrometer scale bar is provided in the confocal images.

From Figure 1, it is evident that both synthesis methods (flask and microfluidic chip) yield similar outcomes. In both cases, particles are formed with diameters around 4 μm. Although particles produced through the microfluidic route exhibit a slightly smaller average diameter (3.5 μm compared to 4.1 μm for flask synthesis), their size distribution is notably broader (2.3 μm for microfluidic versus 1.6 μm for flask synthesis). This variation might arise due to a non-uniform mixing of reagents during their transport along microfluidic channels. As can be seen from Figure 1a,b, the CaCO_3_ particles were predominantly represented by spherical form. A minor fraction of the particles was characterized by a cubic shape (Figure 1a,b, Region 2 and 3). The analysis of the Raman spectra of the samples synthesized in the bulk and microfluidic chip, as depicted in Figure 1c, revealed characteristic single peaks at 1080 and 1090 cm^−1^, which corresponded to the vaterite form of CaCO_3_, as well as the 1085 cm^−1^ peak that corresponds to the calcite form of CaCO_3_[37]. Singlet peaks at 1080 and 1090 cm^−1^ arise from the aginternal mode due to the $\nu_{1}$-symmetric stretching mode of the carbonate ion. The $\nu_{4}$ in-plane bending mode of carbonate can be found at 712 cm^−1^ (calcite) and 749 cm^−1^ (vaterite). Finally, both samples exhibited peaks below 300 cm^−1^, which corresponded to the translational and rotational lattice modes. Notably, while microfluidically synthesized CaCO_3_ microparticles possess a relatively wide size distribution, they also exhibit the potential for a further diameter reduction, which lies in a precise control of the reagents’ reaction time. However, the absence of specific external reaction initiators leaves no alternative but to reduce the concentration of reactant molecules in the particle growth region of the mixture to decrease the reaction rate and/or completely halt the reaction [38]. Therefore, at the next step of the study, we evaluated the particle growth dynamics depending on the concentration of reactant molecules in the precursor mixture. For this purpose, immediately after mixing the reactants in the flask and before precipitating the resulting particles using centrifugation, the precursor mixture was diluted 5 and 10 times. The concentrations of the synthesis reactants and the particle precipitation protocols from the reaction mixture are provided in Section 2.5, Materials and Methods. Figure 2 displays the confocal microscopy images of the resulting particles and their size distribution, which were deposited on glass slides at various degrees of precursor mixture dilution before precipitation.

**Figure 3:**
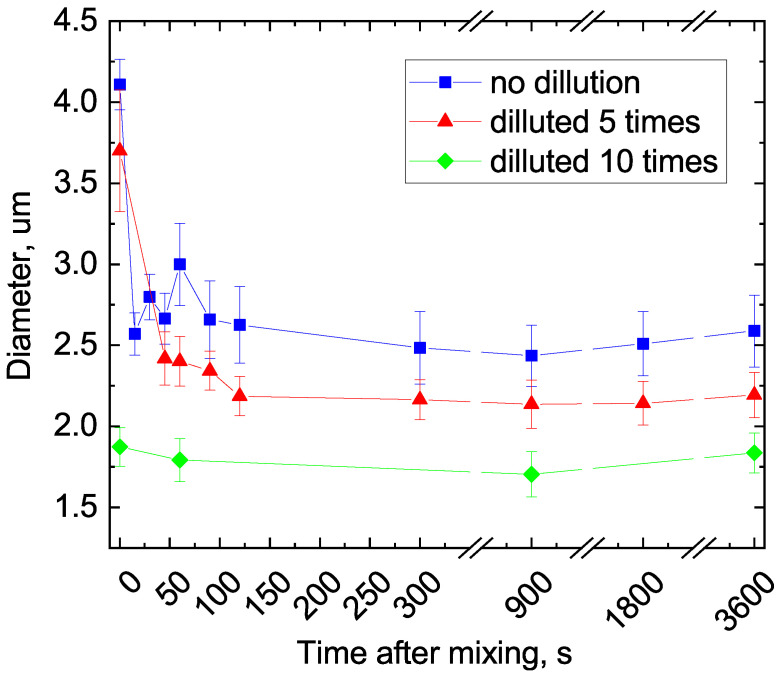
Dependencies of the calcium carbonate microsphere sizes on the time after mixing and the degree of precursor mixture dilution (undiluted—curve with blue squares, diluted by factors of 5—curve with red triangles, and diluted by factors of 10—curve with green diamonds).

Analysis of the calcium carbonate particle size distributions, as depicted in Figure 3b, led to the conclusion that the precipitation of particles from the reaction mixture without dilution allows the formation of particles with a diameter of ∼4 μm and a width distribution of 1.5 μm. Dilution of the reactant mixture by a factor of 5 and 10 led to a reduction in the particle diameter to 3.7 μm (size distribution—1.5 μm) and 2.9 μm (size distribution—0.85 μm), respectively. The increase in the size of calcium carbonate particles by a factor of two when the reaction mixture was diluted by two-fold before precipitation was likely attributed to the additional reagent mixing along with relatively moderate concentration dilution. Conversely, a further reduction in the precursor concentrations in the reaction mixture resulted in an additional reduction in particle size compared to the original undiluted mixture. This particle growth dynamic was likely caused by a sharp change in the pH level from 9.6 to 7 in the reaction mixture. Such a transition from alkaline to neutral conditions leads to particle size reduction [39]. Based on the data obtained, it can be inferred that dilution of the reaction mixture by ten or more times is an effective strategy for not only halting the reaction of calcium carbonate particle growth, but also for reducing their final size. Additionally, we investigated the kinetics of calcium carbonate particle growth as a function of the dilution degree in the reaction mixture prior to precipitation. Figure 3 presents the results of the analysis of the average size of the obtained calcium carbonate microspheres, which were determined depending on the degree of precursor mixture dilution and time after mixing.

As observed from the curves presented in Figure 3, the dilution of the reagent mixture to 2, 4, and 10 times effectively decreased the size of the resultant CaCO_3_ particles up to three times the original size (1.7 μm for a 10 times diluted solution against 4 μm particles of an undiluted reagent mixture). A consistent trend of gradual particle size reduction over time after mixing was evident across nearly all samples and can be attributed to a redistribution of free ions within the precursor mixture, which induces calcium carbonate crystal rearrangement [40]. Based on the data obtained, the dilution of the reagent mixture after precursor mixing and prior precipitation from the residual reagent molecules emerged as a significant synthesis parameter. Additionally, the data presented in Figure 3 show that the implementation of the resting time between dilution and centrifugation further decreases the particle’s diameter. For the precursors diluted by less than an order of magnitude, a resting time of approximately 100–120 s resulted in a reduction in the particle size by 20–30% (from ∼4 μm to ∼2.3 μm). Based on these data, we can expect the precursor mixture transferred from the microfluidic chip to an external flask for further precipitation not to result in uncontrolled particle size growth. The implementation of an efficient mechanism for a partial and/or complete inhibition of calcium carbonate particle synthesis is crucial when transitioning from bulk synthesis to microfluidic chips with high levels of automation.

### 3.2. Reagent Mixing Efficiency in Microfluidic Chips

**Figure 4:**
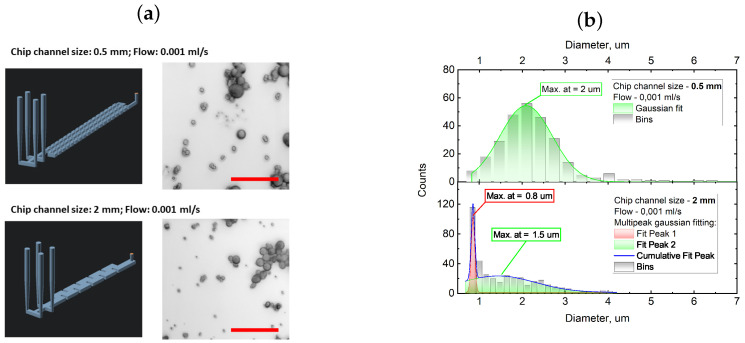
(**a**) Images of the synthesized CaCO_3_ particles with different flow speed and chip channel widths taken by confocal microscopy. (**b**) Size distribution of the synthesized CaCO_3_ particles synthesized with different chip widths (0.5 and 2 mm for the upper and bottom panels, respectively) and a reagent flow speed of 0.001 mL/s.

In the preceding section, we explored the impact of precursor mixture dilution on the diameter of precipitated particles. The established interaction provided the basis for formulating a synthesis protocol for calcium carbonate particles within the microfluidic chips. In this protocol, the final particle size was predominantly influenced by the geometric parameters of the chip and the fluid flow velocities within it. To implement a microfluidic synthesis, two microfluidic chips were fabricated using MSLA 3D printing, as detailed in Section 2.3 of the Materials and Methods. Both chips maintained an inner volume of 100 μL, with one chip having a cross-section of 0.5 mm^2^ and the other 2 mm^2^ (the schematic of the used microfluidic chips is presented in Figure 4a). Initially, we investigated the impact of directly transferring the devised synthesis protocol from a flask to a microfluidic chip on the physical properties of the particles. For this analysis, reagents were introduced into the microfluidic chip at a flow speed of 0.001 mL/s, thus ensuring that the reaction time of the reagent mixture matched the 100 s employed in the flask synthesis discussed in the previous section. Figure 4 illustrates the confocal images of the synthesized particles along with their size distributions.

The analysis of CaCO_3_ particle size distributions, as presented in Figure 4b, revealed the synthesis of a polydisperse ensemble using both microfluidic chip designs. Notably, the size distribution significantly varied between the two chip designs. The 0.5 mm^2^ cross-section chip exhibited a broad distribution of micron-sized particles around 2 μm, while the 2 mm^2^ cross-section chip displayed two distinct peaks at 0.8 and 1.5 μm (Figure 4b, bottom panel). It is noteworthy that the submicron particles demonstrated a greater uniformity compared to their micron-sized counterparts.

The calculation of the Reynolds number for the 3D-printed microfluidic channels used in this work showed that the reagent flow should be laminar with a Re(thin channel) of 2.27 and a Re(wide channel) of 1.14. This leads us to a conclusion that reagent mixing in 3D-printed microfluidic channels occurs mainly through diffusion. Calculation of the Peclet number shows that advection plays a more prominent role in the case of channels with a cross section of 0.5 mm^2^, which equals 1570; meanwhile, for a wider microfluidic channel, this parameter is two times lower and equals 875. Based on these data, we can conclude that chaotic advection plays a major role in the reagent mixing in channels with a smaller cross section, while diffusion is a major mechanism for reagent mixing with wider microfluidic channels [41,42,43,44]. However, the observed polydispersity and presence of distinct ensembles in synthesized CaCO_3_ particles, both submicron- and micron-sized, may be attributed to two distinct regimes of reagent mixing within wide microfluidic channels. This can be explained with a time-resolved analysis of the flow characteristics inside of microfluidic channels.

At first, chaotic advection and vortex generation occurs as the reagent solution traverses the stagnant media within the microfluidic channels. This flow regime arises from the interaction of reagent molecules, where it moves at high flow speeds with immobile water molecules pre-existing in the microfluidic chips before CaCO_3_ particle synthesis. Following a brief duration, the chaotic advection regime transitions to a diffusion mixing of reagent molecules, propelling all molecules within the media toward the outlet. In this regime, a thin area near the center of the microfluidic channel exhibits significant variations in the synthesis parameters, especially in the concentrations of CaCl_2_ and Na_2_CO_3_ salts and their stoichiometric ratio. These factors have been previously shown to significantly influence the growth of CaCO_3_ crystals [30].

**Figure 5:**
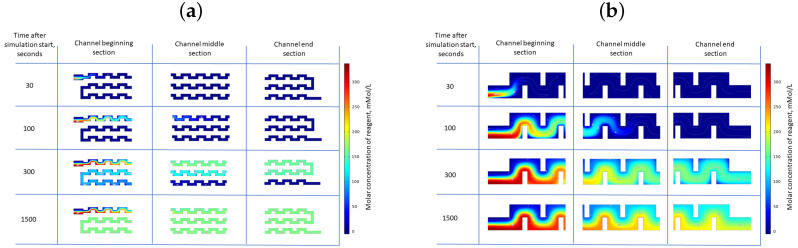
CFD simulation of the reagent mixing in different microfluidic chip sections for a channel cross section of 0.5 mm^2^ (**a**) and 2 mm^2^ (**b**) at different time frames after the start of the pumping cycle.

During the chaotic advection regime, the uncontrollable mixing of the reagent solution across the microfluidic channel resulted in the formation of relatively small micron-sized particles with a broad size distribution. In contrast, the diffusion mixing regime near the center of the microfluidic channel promoted the formation of submicron particles due to an elevated rate of nucleation. As the rate of nucleation increased and the critical nucleus size decreased, the stable nucleus formation prevailed over the subsequent growth, thus following Ostwald’s rule of selection. This shift was attributed to an escalation in supersaturation, thereby leading to a higher rate of viable nucleus formation. To support this hypothesis, computational fluid dynamic (CFD) simulations of reagent mixing were conducted for two distinct microfluidic chip configurations. These are depicted in Figure 5, where the CaCl_2_ reagent concentration during the synthesis of CaCO_3_ particles in microfluidic chips with cross-sectional channel areas of 0.5 and 2 mm^2^ is illustrated.

As shown in Figure 5a, the CaCl_2_ reagent distribution within the microfluidic chip appeared predominantly homogeneous after 300 s of continuous reagent pumping. However, a concentration gradient near the main inlet was noticeable at the initiation of the reagent pumping, which persisted throughout the simulation period. It is crucial to emphasize that this gradient remained stable over time, in contrast to the simulation depicted in Figure 5b, which represents a chip with a wider channel. In the case of wider channels, a gradual transition from the chaotic advection mixing during the initial phase of the simulation was observed, which was followed by the progressive establishment of diffusion mixing at the center of the channel after 300 s of continuous pumping. This behavior aligns with the previously posited assumption where a chip with a smaller cross-section primarily operates in an advection mixing regime, thus leading to the synthesis of micron-sized particles with a broad size distribution (Figure 4b, top panel). Conversely, a chip with an increased cross-section produced two distinct size ensembles of submicron- and micron-sized particles. The former was synthesized along the center of the channel where a high concentration of both reagents was present, while the latter was synthesized near the channel walls. It should be noted that concentration fields for Na_2_CO_3_ in relation to the CaCl_2_ reagent solution behaved virtually identical to CaCl_2_.

To empirically validate the occurrence of the two distinct regimes of reagent flow within a microfluidic chip resulting in such behavior, a series of syntheses were conducted in both chips. In these syntheses, the volume of the reagent solution exiting the microfluidic chip was incrementally increased, starting from 50 μL up to 1000 μL. After traversing the internal volume of the chip, the sampled precursor mixture, along with the formed particles, underwent a ten-fold dilution with distilled water, and it was subsequently subjected to two washes to eliminate unreacted reagent molecules. The parameters for the synthesis of calcium carbonate microspheres in this experiment were the same as the ones described in Figure 4.

**Figure 6:**
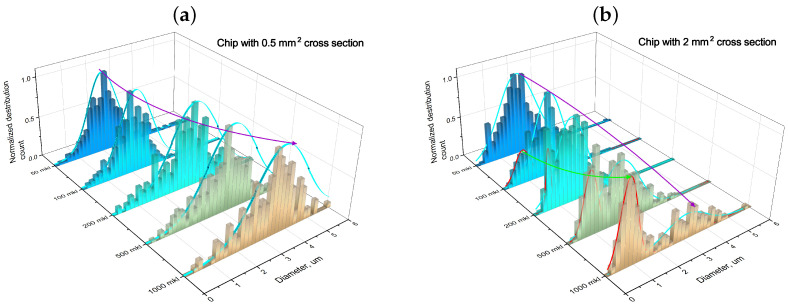
CaCO_3_ size distribution after synthesizing in the microfluidic chip with a channel cross section of 0.5 mm^2^ (**a**) and 2 mm^2^ (**b**), which were dependent on the amount of reagent mix volume that came out of the chip.

Figure 6 presents particle size distribution plots of the calcium carbonate microspheres synthesized in microfluidic chips with channel cross-sections of 0.5 and 2 mm^2^ and varying amounts of exiting reagent mix.

As illustrated in Figure 6, a gradual increase in the volume of the reagent mixture exiting the outlet of the microfluidic chip resulted in a discernible alteration in the size distribution of the synthesized CaCO_3_ particles. Specifically, a slow emergence of a second, narrower peak in the submicron range was observed, with a maximum at 0.8 μm in addition to the original broader peak. We posit that this progression may be attributed to the previously discussed presence of two reagent mixing regimes, which gradually transitioned from turbulent to laminar, influencing the polydispersity of CaCO_3_ particles.

**Figure 7:**
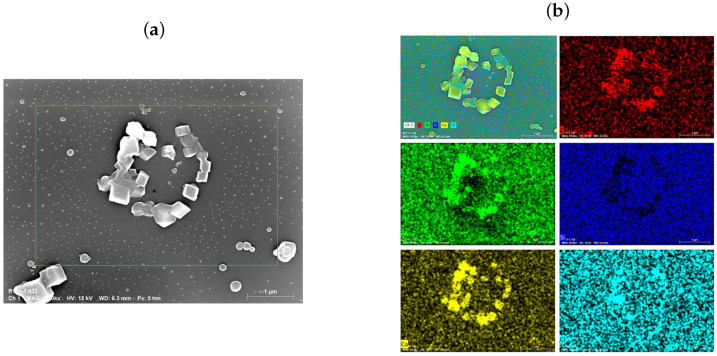
SEM (**a**) and EDX (**b**) images of theCaCO_3_ submicron particles deposited onto a Si wafer. The colors on the EDX images represent the recorded signal from various elements as follows: red—carbon; green—oxygen; blue—silicon; yellow—calcium; and cyan—chloride.

Throughout the synthesis of CaCO_3_ particles in the microfluidic chips, a submicron ensemble was formed. According to the size distribution dependencies presented in Figure 4b and Figure 6d, submicron CaCO_3_ particles constituted a substantial portion, ranging from 50% to 80% of the total number of particles. Notably, a considerable quantity of submicron particles was produced using the microfluidic synthesis method, thus approaching the diffractive resolution limit of the confocal microscope (∼200 nm). To facilitate a more comprehensive analysis, samples of the submicron particles were subjected to investigation using scanning electron microscopy (SEM) and energy-dispersive X-ray spectroscopy (EDX). Figure 7 presents the SEM and EDX images obtained for the nanoscale CaCO_3_ particles synthesized within a microfluidic chip.

As depicted in Figure 7a,b, the particles synthesized within the microfluidic chip exhibited a considerable presence of nanosized particles ranging around 50–90 nm in diameter. Elemental analysis using energy-dispersive X-ray spectroscopy (EDX) confirmed the composition of these particles, thus revealing the presence of calcium, carbon, and oxygen atoms, with slight traces of chloride. Notably, while the micron-sized CaCO_3_ particles predominantly adopted the vaterite form, the submicron particles exhibited a more prevalent occurrence of the calcite form. This phenomenon may be influenced by residual CaCl_2_ reagent traces (detected on the particles), which were not entirely removed before the deposition of particles on a wafer. The presence of these residual reagent molecules could have induced the recrystallization of particles from the vaterite to the calcite form [45].

The data presented in this section serve to validate that the utilization of 3D-printed microfluidic chips with relatively wide channel diameters can yield high-quality submicron and even nanoscale particles. The findings affirm that the microfluidic synthesis of calcium carbonate microspheres is not only achievable, but also surpasses classical flask synthesis in terms of control. Notably, the microfluidic-produced calcium carbonate particles exhibit a submicron size, a highly desirable characteristic for potential applications in biomedicine. Furthermore, this study demonstrates that microfluidic chips can be directly fabricated using additive manufacturing methods, thus eliminating the necessity for traditional soft lithography.

## 4. Conclusions

This investigation delves into the intricacies of calcium carbonate synthesis by employing the microfluidic approach. The use of 3D-printed microfluidic chips in our experiments underscored their appropriateness for achieving precision and control in CaCO_3_ particle synthesis. Our endeavor to achieve smaller particle sizes, which are obtained through the modification of both synthesis and post-synthesis procedures, unveiled a nuanced equilibrium between reducing the particle size and maintaining synthesis reproducibility.

The comprehensive analysis of particle size distribution, coupled with the investigation of varying reagent volumes exiting the microfluidic chip, indicates a noteworthy alteration in the size distribution of the synthesized particles. The emergence of a second, narrower peak in the submicron range suggests a nuanced relationship between the reagent flow dynamics and particle size distribution. The observed transition from chaotic advection to diffusion mixing regimes aligns with the proposed hypothesis of two distinct mixing regimes affecting the polydispersity of CaCO_3_ particles.

Furthermore, the microscopic examination, along with the EDX analysis, reveals the presence of a substantial quantity of nanosized particles (50–90 nm in diameter) within the synthesized particles. Notably, this study highlights the impact of residual reagent traces, particularly CaCl_2_, on the crystalline forms of the particles synthesized. The shift from a predominantly vaterite form in micron-sized particles to a calcite form in submicron particles suggests the potential role of residual reagents in triggering recrystallization.

Overall, the findings affirm the efficacy of microfluidic synthesis in producing high-quality submicron and nanoscale particles, thereby demonstrating superior control compared to classical flask synthesis. The submicron size of microfluidic-produced calcium carbonate particles holds promise for applications in biomedicine. Moreover, the use of 3D-printed microfluidic chips with wide channel diameters presents an innovative approach, thus eliminating the need for traditional soft lithography in chip fabrication. This research contributes valuable insights to the understanding and optimization of microfluidic synthesis processes for the controlled production of submicron and nanoscale particles.

## Figures and Tables

**Table 1:** Parameters of the precursors and microfluidic setup for the CFD calculations of CaCO_3_ reagents mixing.

Parameter	CaCl_2_	Na_2_CO_3_
Viscosity, mPa×s	1.0016	
Density, g/mL	1.0279	1.0207
Diffusion coefficient, m^2^/s ×10^−9^	1.134	1.11
Concentration, mole/m^3^	330	
Temperature, °C	25	
Volumetric flow rate, mL/s	0.001	

## Data Availability

The original contributions presented in the study are included in the article, further inquiries can be directed to the corresponding author.

## Abbreviations

The following abbreviations are used in this manuscript:

EG	Ethylene glycol
MSLA	Masked stereolithography apparatus
DLS	Dynamic light scattering
SEM	Scanning electron microscopy
